# On the Treatment of Cholera Infantum

**Published:** 1858-08

**Authors:** Robert A. Gholson

**Affiliations:** Petersburg, Va.


*On the Treatment of Cholera Infantum.* By Robert A. Gholson, M. D. of Petersburg, Va.

*Therapeutic Treatment.*

I shall first consider the treatment proper in the ordinary mild form of the disease, and afterwards that which may be necessary in its graver, more irregular and complicated types.

In the simple, mild form of cholera infantum, as it generally presents itself, we have the follicular affection as the chief, if not the only pathological element, manifesting itself usually in excessive secretion, leading to increased peristaltic action, vomiting, and purging, without much febrile disturbance of the system. There are two extremes, which the practical physicians should avoid, in his views of the nature and treatment of this profuse follicular secretion. One is the hypothesis, which views this secretion in the light of an effort on the part of nature to deplete and relieve an engorged and inflamed organ, and therefore salutary. The other is the opposite error, which regards this morbid excessive secretion as the disease itself, against which our remedies should be principally if not exclusively directed. There can be but little doubt, I think, that this excessive secretion is connected more with a relaxed than an inflamed condition of the follicles, and is often a positive evil in itself, tending not only to prostrate the forces of the general system, but to irritate and inflame the digestive mucous membrane, and aggravate the morbid condition of the follicles, upon which it depends. But there can be as little doubt, on the other hand, that this profuse secretion is but a *symptom* of the disease—a symptom, too, which is not *always* present, and that when it is observed, we may often arrest it without curing the disease. The principle indication in the treatment of this follicular affection would therefore seem to be to alter or subvert the morbid condition of the follicles, upon which the secretion depends, and at the same time to arrest, or at least restrain, within safe and reasonable bounds, the secretion itself.

I will here remark, "in limine," that in our treatment of the follicular disease, we should ever bear in mind the sympathetic disturbance in the functions of the great emunctories—the skin, the liver and the kidneys—and that as long as this disturbance remains, it will be very difficult permanently to relieve the follicular disease; and that when we do, it is often replaced by a disease of some other organ, far more formidable. While we endeavor, therefore, to arrest the follicular affection, and the copious discharges from the stomach and bowels, we must be careful at the same time to re-establish the healthy functions of these organs.—This caution will be especially necessary after the disease has continued some time, and the system has been accustomed to a flux or drain from the digestive mucous membrane. Here a sudden arrest of the discharges will be very apt to be followed by an inflammation or dropsy in the bowels, brain or some other part of the body, especially when there is any functional inactivity of the emunctories.

With these preliminary remarks in relation to the general principles which should guide us in the management of the disease, let us now enquire what are the remedial means which at the present day, and in the present state of our knowledge, are most reliable in controlling this follicular disease. Confining ourselves at present to a consideration of the internal remedies, there are two principal systems of treatment which at present divide the profession both in this country and Europe. One is the soothing, sedative, alterative course—the other is the perturbating, emetic and cathartic treatment. The latter treatment in this country is connected very much with that all pervading *hepatic pathology*, which even now so materially influences the treatment of nearly all our diseases. In Europe, especially in France, its advocates rely upon it not to *disgorge* or *emulge* the liver, but by its perturbating and revolutionary influences, to alter and subvert the morbid condition of the muciporous follicles.—However well it may be borne in the more temperate climate of Europe, and perhaps in the more northern portions of this country, I regard it as an unsafe and hazardous practice among us, leading *occasionally* to speedy and prompt collapse, and *very frequently* to inflammation of some portion of the digestive mucus membrane. With the exception, therefore, of a few rare cases, in which an emetic or cathartic may be necessary to clear the stomach and bowels *at once* of offensive and irritating ingesta or secretions, I think we had better dispense with this class of medicines in this form of the disease, and depend upon those of a sooth-ing, sedative and alterative character. Of this latter class of remedies, *sugar of lead* and *calomel* stand pre-eminent. The sugar of lead espe-cially is the great remedy in all stages of this form of the disease, where the discharges are watery and profuse, and require checking. Possessed of the singular virtue which we find in no other article of the materia, medica, of combining the properties of both a reliable sedative and as-tringent, it seems to be peculiarly suited to this form of cholera infantum. Improperly classed until recently among the astringents, its valuable se-dative powers are just beginning to be recognized. To Dr. Condie of Philadelphia is the profession principally indebted for bringing it into use in the treatment of the *early* or *choleric* stage of this disease. My own experience with it, like that of almost every physician who has given it a trial, is so encouraging and satisfactory, that I consider it as the most valuable remedy we have in all its stages, where the discharges are pro-fuse and watery, where there is not much inflammation on the one hand, or prostration on the other. No remedy we possess exercises such a prompt and happy control over that *extreme irritability of the stomach*, which is such an harassing and exhausting symptom in the commence-ment of this disease. For this purpose it should be given at first, while the stomach is thus irritable, rejecting almost every thing taken into it, in very small doses. A very eligible mode of administering it, is that recommended by Dr. Condie, in the following form: ℞ Aqua. puræ, ℥j; acet. plumbi, gr. v; acid acet. imp. m, v.; sach. puræ, ℥iij. M. One teaspoonful to be given every hour, or until the vomiting is suspended.

But sugar of lead is inferior to the *calomel*, the other great alterative in this disease, in one respect. It has no direct power over the biliary secretion, which is generally deficient or perverted in this disease.—Hence, for its well established power over the liver as well as the folli-cular disease, calomel should be administered alternately with the sugar of lead, in small doses, say from one-twelfth to one-sixth of a grain, and persevered in until its effects are displayed, or we are satisfied of its inu-tility. These are the two great internal remedies, upon which we must principally rely in this, the choleric stage of the disease. There are, however, various auxiliary remedies, which particular conditions of the stomach or general system may demand. Thus, when the discharges indi-cate *acidity*, we will derive considerable benefit from the administration occasionally of a few mouthfuls of cold carbonic acid water, in which a small portion of bicarb. of soda has been dissolved; or if this is not convenient, of gum arabic and lime water. In an opposite *alkaline* condition of the stomach some *acid* will be required. In such cases *strong lemonade* was a favorite remedy with Professor Chapman. Where there is a tendency to *collapse*, we must resort to some stimulant, as the chamhor julep, mint julep, Hoffman's anodyne,"paregoric &c. But the irritability of the stomach is often so extreme, that along with thes remedies to be administered per orem, we must use others, addressed to the skin and rectum. The *tepid* or *warm bath* will generally exercise a happy influence in restoring the equilibrium of the circulation, and allaying nervous irritability, and for this purpose should be used from time to time through the whole course of the disease. A mustard plaster or mustard poultice should also be applied to the stomach, while the extremities may be bathed in a mustard bath, and the spine rubbed with some stimulating liniment. If, notwithstanding the use of these means, the irritability of the stomach persists, we had better resort at once to an *anodyne enema*, consisting of three drops of laudanum (for a child one year old,) mixed with a few teaspoonfuls of some mucilaginous fluid. This rarely fails in checking the vomiting, quieting the irritabilty of the system, and securing a refreshing interval of sleep or repose, and may be repeated "pro re nata."

Under the prompt and judicious use of the above means, the choleric stage usually passes off in the course of a few days, when the disease presents itself in the form of diarrhœa. The subsequent treatment in this, the diarrhœal stage of the disease, will of course depend upon the character of the discharges and condition of the general system. If the sugar of lead and calomel have acted happily in regulating the secretions from the muciparous follicles and liver, and little else is left but an undue peristaltic action of the bowels, nothing will be required to complete the recovery but a well regulated diet, the tepid bath, and perhaps some cordial laxative, combined with or followed by an anodyne; and for this purpose, nothing answers better than the spiced syrup of rhubarb and paregoric, or if we are afraid of any preparation of opium, the hyoscyamus which is a remedy of inestimable value in the affections of children. A few drops of the paregoric or the henbane tincture may be combined with a half teaspoonful of the spiced rhubarb, and repeated from time to time as may be necessary. When we have reason to suppose that the morbid secretion or ingesta unduly are retained in the bowels, we may ıender the rhubarb a little more laxative by the addition of a little calomel or magnesia. When the discharges present a greenish aspect, resembling often chopped spinnage, indicating probably an excess of acid, some *antacid*, as the bicarb of soda, or magnesia, or prepared chalk, should form one of the ingredients of our prescriptions. Where the discharges are *clay-colored* or *whitish*, showing a deficiency of bile, we must continue the use of some preparation of mercury, as the calomel, or the blue powder, the hydrarg. c. creta; and where they continue watery and profuse, as in the choleric stage, there is no remedy so reliable as the sugar of lead. The stomach being retentive, the calomel and sugar of lead may now be given in larger doses, and if necessary, in combination, say from a half to one grain of the acetate, with one-eighth to one-fourth of a grain of calomel, mixed in a little spiced syrup of rhubarb. A few drops of paregoric or tinct. of hyoscyamus may, when necessary, be added to this mixture. A favorite prescription with Dr. Condie in such cases is the following: ℞ Calomel, grs. iij; cret. prepar. grs. xxxvj; acet. plumbi, grs. xij; ipp. pulv. grs. iij. F. Ch. No. 12. One of these powders to be given every three hours.

There are very few cases of this *mild form* of the disease, which will not yield in the course of a few days to the above remedies. Should they fail, however, after being given a fair trial, we must then resort at once to the class of *astringents*, and not suffer the disease to degenerate in the chronic form. We had better commence with the *tannic acid*, as it is the purest and nicest of all the astringents, free from all stimulating or tonic properties and generally well borne by the stomach. If the alterative, sedative powers of sugar of lead are still needed, the two remedies may be combined as in the following mixture: ℞. Acet. plumbi, acid tannic, aa. gr, iv; tinct. opii. camph.; sy zingiber, aa. ʒi; aquæ distillatæ, ℥vi. M. One teaspoonful every four hours.

In the more *advanced* stages, however, where the system begins to require support, we must select some other astringent, possessing *tonic* properties, as logwood, rhatany, catechu, &c. I have derived much benefit in such cases from mixtures like the following: ℞. Ext. hematoxy, ʒi; tinct. krameriæ, ʒii; sy. acaciæ, ʒii; aq. cinam. ℥viii. M. One teaspoonful every three or four hours.

If necessary, a little laudanum, paregoric or tinct of hyoscyamus may be added to each dose of the above mixture. At this stage of the disease, where it threatens to become chronic, especially if attended with much emaciation and debility, we must be very cautious in the farther use of *mercury*. No practice is more reprehensible than the indiscriminate and continued use of this powerful antiphlogistic in all stages of this disease. It often lays the foundation of a state of hopeless cachexia, from which no subsequent course of treatment, however judicious and persevering, is effectual in rescuing its victims. In the latter or *subacute* stages of the disease, we may often reap much benefit from various alterative, astringent and tonic remedies, which will be more particularly referred to, under the chronic form of the disease. It often happens that however well a particular preparation may answer our purpose at first, the system soon loses its susceptibility to its influence, and it becomes necessary to change it for some other. The above remedies, however, are those which I have usually found most effectual in the management of the ordinary mild form of this disease.

But cholera infantum does not always present itself in the *mild form,* we have been thus far considering. The simple follicular affection is often complicated both in the choleric and diarrhœal stage, with other pathological conditions, which demand corresponding modifications of treatment.

*Inflammation* of some portion of the digestive mucous membrane is liable to supervene at any time in this disease, but especially in the diarrhœal stage, in the form of *entero-colitis*, decided *dysenteric* symptoms setting in, often in the course of a day or so, and rapidly resulting in a tender and swollen abdomen, a predominance of mucus or blood in the discharges, attended with more or less tormina and tenesmus, and continued symptomatic fever Such symptoms should be promptly met, whenever they present themselves, with the great antiphlogistics—*blood-letting*, *mercury*, *cold water*, *revulsives*, *&c.*, which should be used with a vigor commensurate with the grade of the inflammation, stage of the disease and resources of the system. *Leeches* should be applied to the epigastric or iliac region, or around the anus, as the symptoms may indicate, followed by emollient poultices, and subsequently warm anodyne fomentations, rubefacients and blisters. At the same time, *calomel*, either alone or combined with ipecac., hyoscyamus or opium, as the symptoms may indicate, should be given a fair trial. In many such cases, however, when the inflammation is confined chiefly to the lower bowels, in the form of *dysentery*, with more or less tormina and tenesmus, we will reap more benefit from *oleaginous mixtnres*, containing more or less laudanum. When the discharges are of that greenish character, which indicates acidity, we will probably derive more benefit from about half a teaspoonful of spiced syrup or rhubarb, with a few grains of carbonate of soda or magnesia, and a few drops of tincture of hyoscyamus, repeated every four hours. In all cases, however, when the tormina and tenesmus are great, we must use from time to time *anodyne enemata*. When the inflammation is higher up the canal, and is yet in the acute state, and no symptoms of prostration, *cold water enemata* will prove of valuable aid. In this inflammatory type, the child's diet should be confined chiefly to mucilaginous drinks, which should be rendered slightly alkaline by the addition of a little bicarbonate of soda or lime water. *Ice*, too, should be given it to suck, confined in a thin gauze bag. This inflammatory form of the disease, when the acute stage begins to pass off, often leaves behind a subacute affection of the large intestine, confined chiefly to the rectum, attended with troublesome tormina and tenesmus. Here we must give sedative and astringent as well as anodyne injections. We should first commence with the sugar of lead, as being the most soothing and sedative of this class—next the sulphate of zinc, and then the nitrate of silver. Two or three grains of the acetate of lead or sulphate of zinc, or about half a grain of the nitrate of silver to two tablespoonfuls of mucillaginous fluid, mixed with a few drops of laudanum, will constitute an enema of suitable strength. I need not say that the return of the enema should be prevented by external pressure.

But there are other sources of danger in cholera infantum, besides inflammation of the *stomach* and *bowels*, symptoms referable to the *nervou* system or *brain*, that frequently present themselves and demand prompt attention. This is very apt to be the case when *teething* plays a predominent part in bringing on the disease. Here the disease will be ushered in with great nervous irritability and restlessness, attended with hot, swollen and inflamed gums, a flushed face, an excited eye and hot head, while the stomach and bowels may be *comparatively quiet*. In such cases, especially if attended with much vascular excitement, we must incise the gums, leech the temples or mastoid processes, apply iced cloths and bladders to the head, revulsives to the extremities, give a tepid bath, a carthartic dose of calomel, or establish revulsion in the lower bowels by a cathartic enema. In such cases, the application of *blisters* behind the ears, or to the epigastric and abdominal regions, when they are the seat of the inflammation, is a common practice, and recommended by the highest authorities. I must confess, however, that I have never been partial to the use of blisters in the diseases of infancy, and always avoid them whenever I can. I have found them more frequently *co-irritating* than *counter-irritating* agents, and believe there are but few cases, in which we cannot reap as much benefit from rubefacients as blisters. But in the aggravated cases of this disease we should leave no means untried which gencral experience sanctions. I need not say, however, that when deemed advisable, they should be used and watched with much caution, should never be allowed to remain longer than to redden the skin, when vesication should be completed by a soft light poultice. When there is much nervous irritability, and we fear the effects of the blister, the cerate, before being spread, should be rubbed up with a small quantity of camphor and opium, say about three grs. of camphor, to one gr. of opium, for a plaster some three or four inches square, and before it is applied to the skin, should *always* be covered with fine gauze. The neglect of this latter precaution is a dirty as well as hazardous practice, leading often to great irritation, and sometimes strangury. But while we must not hesitate to resort promptly to blood letting, mercury, cold water, cathartics and revulsives, whenever inflammation threatens to tnvade the brain aud its membranes, we must be careful in avoiding unnecessary depletion in a disease, one of the most striking featuaes of which is *rapid emaciation and debility.* We should constantly bear in mind, too, the *anatomical peculiarities* of the infantile system, the excessive development of its spinal nervous system, and remember that very considerable disturbance in the nervous system, such as we would justly regard in the *adult* as denoting grave cerebral lession, very often occurs in the *infant* without any intra-cranial disease. Whenever, therefore, suspicious nervous symptoms present themselves in the progress of this disease, we must be very careful to interpret them correctly. My observation leads me to believe that many children in this disease are sacrificed by a depleting line of treatment, based upon a groundless apprehension of encephalic disease. A child, at any period of the disease, becomes irritable, restless and excitable—it starts and becomes alarmed from the slightest noise—anon it falls into an imperfect slumber, in which we discover some twitchings of the tendons, or even of the muscles of the face—presently it starts from its sleep in screams of terror and alarm, with perhaps some momentary flush of cheek and heat of head. We fear disease is invading the brain. We immediately order *loss of blood, cold to the head and a cathartic.* But the nervous symptoms *increase,* and the child finally goes into convulsions, and, may be, dies. Here the *tepid bath,* followed by a composing draught or anodyne enema, with cold to the head, and a dark, cool and quiet room would have restored the equilibrium of the circulation, quieted nervous irritability, and arrested at once the tendency to convulsions.

Again: in the latter stages of the disease, the child will fall into a state of *drowsiness and stupor,* in which it will make that piteous *wail* or *moan,* so sad to a mother's heart—the discharges, perhaps, have ceased, or notably diminished, and we fear that by a kind of metastasis or translation, the head has become affected. We change at once our plan of treatment—we stop the use of our astringents and tonics, and resort to calomel, cold, and it may be, leeches to the head. But the stupor increases, and passes rapidly into coma and death.

Here the explanation of these symptoms is not *inflammation of the*

*brain*, but, on the contrary, debility; flagging of the dynamic nervous energies; cutting off the supply of nerve power, both to the bowels and the brain; arresting the secretions from the one, and sinking the other in the stupor of prostration. Nothing but the prompt and timely support of the system by suitable stimulants can save the child at such a critical juncture. In all such cases, when our suspicions of encephalic disease are excited, we must examine critically into the condition of the system; the state of the circulation; the condition of the skin; the color of the face, heat of head, resources of the system, &c. The state of the mind and feelings, too, must not escape our observation. Even at the tender age of infancy, much can be learned of the state of the brain, from the display of its intellectual functions. If suspicious and ugly nervous symptoms present themselves, and we find the pulse firm, quick and excited, the skin hot and dry, the cheeks flushed, the head hot, the blood vessels about the neck, temples and fontanelle throbbing, and especially, if the child lies sad and listless, never catching its mother's eye and showing the happy and contented look of recognition, then we can hardly err in adopting a depleting line of treatment.

But of all the grave *inflammatory* forms which cholera infantum sometimes assumes, the *epidemic type* is the most malignant. This form seems to be connected with some special condition of the atmosphere, the same perhaps as in this section of country is concerned in the production of our epidemic dysentery, to which indeed it bears a striking analogy—both diseases often pervading a community at the same time—both affecting principally the large intestine, running the same rapid and fatal course and carrying terror and death into every family they visit. Its mode of access or invasion is generally sudden, unheralded often by any premonitory symptoms. A child will be suddenly seized. perhaps while asleep, with the disease; its extremities become cold; its skin pale and shriveled; it moans and gives every evidence of being unwell; soon it awakes, begins to vomit; after a while to purge more or less, reactionary fever sooner or later coming on, attended with hot skin, great thirst, epigastric and abdominal distress; these symptoms gradually ameliorating, and ending in remission much more marked and striking than we generally observe in cholera infantum, and, followed in the course of a few hours by another paroxysm, leading usually to more or less inflammation of the large intestine, evinced by mucous or bloody stools, more or less tormina and tenesmus, a tender and often a swollen abdomen, and often to great sympathetic disturbance in the brain or nervous system.

The safety of the child in such cases will often depend upon the early elimination from the disease of its *malarial* or *periodical* element.—Hence the *sulphate of quinine* should be administered at the *earliest possible period;* and with this view we should attack any condition of the system which may be incompatible with its use, with energy and boldness which would be rash and reprehensible under other circumstances. As soon as the paroxysm begins to moderate we should resort at once to the quinine, which should be given in an enema composed of two grs. of quinine and as many drops of laudanum, mixed in about one tablespoonful or more of any mucilaginous fluid. If the first enema should be well borne, it should be repeated every three or four hours until we think enough has been administered. While we are using the quinine, we may at the same time apply cold to the head, and poultices, rubefacients or blisters to the stomach and boweles. After divesting this form of the disease of its periodical feature, its subsequent management will not differ materially from that proper in other inflammatory forms.

Having briefly detailed the treatment which I have found most successful in the acute stages of this disease, in its diversified forms, I will now say a few words on the management of the disease when it becomes *chronic.* In the great majority of such cases, we have more or less inflammation and ulceration of the digestive mucous membrane and its follicles, seated chiefly in the large intestine, associated with much emaciation and debility. All experience teaches that chronic ulceration of the bowels is but little under the influence of medicines and requires patience and time in healing. While, therefore, we address remedies to the local affection, we must take care to husband the resources of the system until art and nature shall be able to repair the mischief done to the bowels. What shall we say here of mercury and the acetate of lead, the two principal alterative, sedative agents in the more acute stages of this disease? As observed elsewhere, the time for mercury has pretty well passed by, and nothing but the most pressing indications for its use will justify its farther employment. Much more benefit may be expected from the sugar of lead, especially in those cases in which the discharges continue frequent and watery. In all such cases we will reap much benefit from the various vegetable tonic astringents. Besides the logwood, rhatany, kino, &c. recommended in the subacute stages, in the form of chalk juleps, decoctions in either water or milk, of cranesbill, blackberry or dewberry roots, make very eligible preparations and are admirably adapted to such cases. But the class of vegetable astringents is not so well suited to those dysenteric cases, in which the discharges are mucous, gleety or purulent. Here the mineral astringents, alteratives and acids, as well as the turpentines and balsams are more appropriate. All these articles may be profitably combined occasionally with some preparation of opium or hyoscyamus. If the sugar of lead has not been used before, we should commence with that, then try, if necessary, the sulphate of zinc, sulphate of copper, alum, nitrate of silver, &c. These failing, we may resort to the nitric or nitromuriatic acids. These again failing, we may try an emulsion of oil of turpentine or balsm copaiba.— The oil of turpentine is particularly suited to those cases in which there is much flatulent distension of the bowels. When the discharges are acrid, offensive and dark colored, Condie and other authors recommend pulverized charcoal. Condie uses it in the following form: ℞ Carb. ligni. ʒi to ʒij; pulv. rhei. ℈ij; ipec. grs. iv to xij; ext. hyos grs. xij: M. F. Ch. No. 12. One given every three or four hous. When the chronic inflammation and ulceration are confined chiefly to the lower part of the large intestine, the sedative and astringents must be administered, as advised elsewhere, per rectum, The sulphate of zinc has done more good in my hands than any other of this class. We may commence with about two grains, and gradually increase the dose. It may be dissolved in about about one or two tablespoonsful of any mucilaginous fluid, with a few drops of laudanum, administered two or three times a day.

Along with these internal remedies we must not neglect the assiduous employment of the warm bath (which may be rendered stimulating by muetard, salt, &c.), and suitable applications to the bowels, as frictions, rubefacients and blisters.

In all cases attended with much emaciation and debility and loss of appetite, we must endeavor to brace up and invigorate the system by tonics and stimulants. In the selection of a tonic or stimulants, we will of course consult the peculiar requirements of the case in hand. If there is much anemia or degradation of the constitution from the abuse of mercury of any other cause, we will select some suitable preparation of iron, as the iodide of iron or the citrate of iron and quinine, &c. The quinine or the compound tincture of bark will be especially suited to those cases connected with a malarial influence. Often the best thing we can use, where there is much emaciation and exhaustion, is a good article of old brandy. It is generally relished and well borne, and when this is the case, should always be used.

In the long, protracted, chronic cases of this disease in which the energies of the system are worn down and exhausted, as much will depend upon the general regimen as any drugs we may administer. The diet especially requires our attention. The child's life often depends upon its being kept alive for a few days or weeks, until the cool weather of autumn can come to its rescue; and it is all important that we see that it gets all the nourishment from food which its enfeebled digestion will permit. In many of these cases no arbitrary rules of diet can be laid down. Milk of any kind is often not well borne. So with the various farinaceous preparations. Animal teas, broths or juices will frequently suit better—such as a beef, veal or chicken tea, or the juice of bacon ham. Where the child is weaned, or old enough, it may be allowed to suck or eat a mouthful or so of smoked salt herring, or boiled or broiled ham. Of all the articles of diet, however, which is generally most suitable in these inveterate cases, I need not say that the sweet potato in this region of Virginia stands unrivaled. There are few children who are not fond of this delicious and valuable root, and who when suffering from the chronic forms of this disease, do not prefer it to any thing else we can give them. The opinion is pretty generally entertained among our physicians, not I think without good foundation, that besides the highly digestible and nutrient qualities of the sweet potato, it acts remedially in this disease, by its soft, emollient, shielding influence over the irritated mucus membrane. There are many cases of the disease, in which no medicine seems to do any good, and in which every thing depends upon keeping up the strength of the child until cool weather. In such cases, it is a common practice with the physicians of Southampton and other tide water counties, to dispense generally with all medicines, and put the child upon old apple brandy and sweet potatoes, and generally with the happiest result. But as before remarked, it is often impossible to say beforehand what will agree with our little patients in such cases. They often conceive a fondness for articles of diet which seems very objectionable, but which, upon trial, agree admirably. Hence we will often act wisely in consulting rather the suggestions of nature than the dictates of reason, and remember there is often much truth in the maxim, "Quod sapit, nutrit."—*Virginia Medical Journal.*

